# The Influence of Parent Pairs with Different Genetic Distances on the Genetic Diversity of Offspring in *Strongylocentrotus intermedius*

**DOI:** 10.3390/biology14070745

**Published:** 2025-06-23

**Authors:** Peng Liu, Xuechun Jiang, Hao Guo, Tongshan Jia, Shuaichen Wu, Fanjiang Ou, Wenzhuo Tian, Lei Liu, Yaqing Chang, Jun Ding, Weijie Zhang

**Affiliations:** Key Laboratory of Mariculture & Stock Enhancement in North China’s Sea, Ministry of Agriculture and Rural Affairs, Dalian Ocean University, Dalian 116023, China; 18864951076@163.com (P.L.);

**Keywords:** *Strongylocentrotus intermedius*, genetic distance, genetic diversity, genetic structure

## Abstract

To identify effective strategies for preserving the genetic diversity of *Strongylocentrotus intermedius* populations, this study employed 15 SSR loci and SSR-seq technology to construct three parental mating groups based on different genetic distances: relatively distant (0.33640), relatively close (0.13051), and mixed (0.29916). These mating groups were used to produce three corresponding offspring populations: the distant group (D), the close group (C), and the mixed group (M). A total of 150 offspring from these populations were genotyped to analyze the effects of parental genetic distance on the genetic diversity of their offspring. The results showed that the observed allele number (Na) in the D and M groups was 4.200 and 4.733, respectively, both lower than the parental family population (FP) group (5.000) but higher than the C group (3.571). The effective allele number (Ne) in the D and M groups was 2.782 and 2.728, respectively, slightly below that of the parental FP group (2.816) but greater than the C group (2.211). Similarly, the observed heterozygosity (Ho) in the D and M groups was 0.496 and 0.488, respectively, both below that of the parental FP group (0.522) but above the C group (0.447). The expected heterozygosity (He) in the D and M groups was 0.586 and 0.579, respectively, slightly lower than the parental FP group (0.595) but higher than the C group (0.487). Additionally, the polymorphism information content (PIC) in the D and M groups was 0.530 and 0.531, respectively, indicating high polymorphism, although slightly lower than the parental FP group (0.546) and significantly higher than the C group (0.438). These findings indicate that the genetic diversity of all the three offspring populations declined to varying degrees compared to the parental population, with the C group experiencing the most severe reduction. In contrast, the D and M groups maintained comparably higher levels of genetic diversity, which were comparable to each other. This study underscores the importance of increasing the genetic distance between parents or adopting mixed mating strategies to sustain genetic diversity in breeding populations. These approaches are recommended for future breeding programs to ensure the long-term conservation and sustainability of genetic resources.

## Simple Summary

Maintaining genetic diversity in farmed sea urchins is essential to prevent health and productivity declines caused by inbreeding. This study tested whether breeding parents with distinct genetic backgrounds preserves diversity in offspring. We compared three strategies: pairing distantly related, closely related, or mixed parents. Offspring from distantly related or mixed parents retained higher genetic diversity, while closely related pairs led to significant losses. Although all offspring showed reduced diversity compared to wild populations, the findings highlight that avoiding close genetic matches and diversifying parent selection can mitigate declines. This approach offers practical solutions for sustainable aquaculture, ensuring healthier populations and long-term industry resilience.

## 1. Introduction

Edible sea urchins constitute a vital global fishery resource, particularly in Asia, the Mediterranean, and the Western Hemisphere [1]. *Strongylocentrotus intermedius*, native to coastal waters of the Russian Far East, eastern Korea, and northern Japan [2], was introduced from Hokkaido, Japan, to China in 1989 by the Dalian Ocean University [3]. Its rapid growth, exceptional gonad yield, short spines, and aquaculture adaptability have established it as the dominant farmed species in northern China [4]. However, the limited founder population size and inadequate characterization of parental genetic relationships in commercial hatcheries have led to inbreeding depression and reduced genetic diversity within cultured stocks. These issues impede the sustainable development of China’s *S. intermedius* aquaculture industry [5]. Consequently, implementing appropriate breeding strategies to maintain offspring genetic diversity is essential for the industry’s long-term viability [6,7].

Studies have shown that breeding strategies play a critical role in shaping the genetic diversity of offspring populations [8,9]. Both family selection and mass selection are designed to accumulate advantageous traits, leading to populations with increased genetic differentiation [10]. However, these selection processes often result in an increase in homozygosity and a decrease in heterozygosity within populations, thereby reducing genetic diversity. For example, Liu et al. [11] observed in their study of *Litopenaeus vannamei* (White shrimp) breeding families that multi-generational selection resulted in heterozygosity loss and increased homozygosity. Such changes elevate the likelihood of harmful gene homozygosity, increasing the risk of inbreeding depression [12], which may compromise the conservation of germplasm resources. Similarly, Cheng et al. [13] compared the genetic diversity of two farmed family populations and three wild populations of *Siniperca chuatsi *(Mandarin fish). Their findings indicated that the genetic diversity of farmed populations was lower than that of wild populations and that farmed populations exhibited greater genetic differentiation. However, the study also revealed that rational parental pairings that avoid inbreeding can effectively preserve the genetic diversity of offspring populations. Fu et al. [14] further confirmed this in their work with *Haliotis diversicolor* (Taiwan abalone), where they avoided mating among full siblings, half-siblings, and close relatives when constructing family populations. As a result, the offspring populations exhibited high genetic diversity and stable genetic structures, ultimately leading to the development of new strains that contribute to germplasm resource conservation. These studies emphasize the importance of controlling parental pairings in family-based breeding programs to minimize inbreeding and maintain genetic diversity across generations [15,16,17]. Moreover, research suggests that parents with greater genetic distances are more likely to produce hybrid offspring with higher genetic diversity [18]. For instance, Han et al. [19] demonstrated a significant positive correlation between the combining ability of 34 full-sibling offspring groups and the genetic distance between parental populations in *Populus tomentosa* (Chinese white poplar). Similar patterns have been observed in aquaculture species. For example, Wei et al. [20] reported a positive correlation between hybrid vigor and genetic distance in F2 and F3 hybrid populations of *Oreochromis niloticus *(Nile tilapia) (♀) × *Sarotherodon melanotheron* (Black-chinned tilapia) (♂). In China, selective breeding of the sea urchin *S. intermedius* has been carried out since 2006. We focused on key traits such as growth rate, gonad quality, and disease resistance as breeding objectives, resulting in the development of two new varieties and several improved strains [21,22,23,24]. Our previous studies demonstrated that family-based selection is more effective than individual selection in maintaining the genetic diversity of breeding populations [25], providing a fundamental basis for germplasm resource conservation in aquaculture breeding programs. Therefore, implementing breeding strategies that account for genetic distance and minimize inbreeding is crucial to preserving genetic diversity and ensuring sustainable genetic improvements in aquaculture populations.

This study seeks to further investigate the effect of parental genetic distance on the genetic diversity of offspring populations, aiming to identify alternative approaches for preserving genetic diversity. The results are expected to offer theoretical insights to support the selective breeding of *S. intermedius*.

## 2. Materials and Methods

### 2.1. Experimental Material

**Figure 1:**
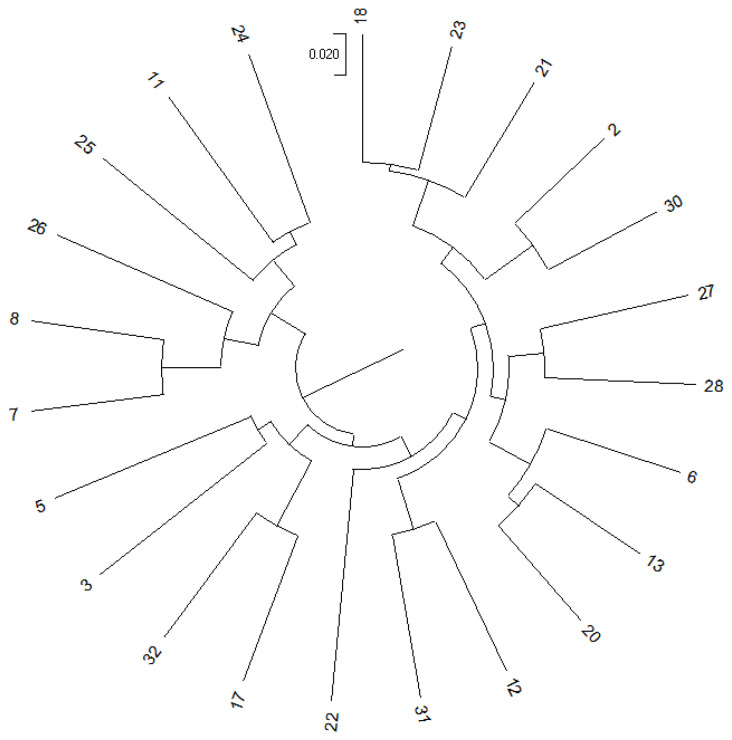
Phylogenetic tree of the 23 parents.

The experimental *S. intermedius* individuals were selected from a selectively bred family population (FP group) cultivated at the Key Laboratory of Mariculture & Stock Enhancement in North China’s Sea, Ministry of Agriculture and Rural Affairs, Dalian Ocean University. A total of 23 individuals were randomly selected as candidate breeding parents. Genetic distances among these parental individuals were measured, and Principal Coordinates Analysis (PCA) was performed. Using the Neighbor-Joining (NJ) method, a phylogenetic tree illustrating the evolutionary relationships of the 23 parental individuals was constructed (Figure 1). Spawning was induced in all parental individuals by injecting 1.5 mL of 0.5 M potassium chloride solution.

Based on the spawning results and the measured genetic distances among the parents, three experimental populations were established: the distant group (D), the close group (C), and the mixed group (M). The D group (D1, D2, D3, D4, D5) was formed using parental pairs with distant genetic distances, with an average genetic distance of 0.33640. The C group (C1, C2, C3, C4, C5) was constructed using parental pairs with close genetic distances, with an average genetic distance of 0.13051. The M group was created by mixing gametes from selected remaining parents to produce a hybrid population, with an average genetic distance of 0.29916. The specific parental combinations used to construct the three experimental populations are systematically documented in the experimental design schema (Table 1).

### 2.2. Extraction of Genomic DNA

Six-month-old offspring from the D and C groups were sampled, with five families selected from each group. Ten individuals were randomly chosen from each family. Additionally, 50 individuals were randomly selected from the M group, resulting in a total of 150 offspring. DNA samples were collected from the 23 parental individuals and the 150 offspring. For the parents, tube feet were clipped and stored at −80 °C. For the offspring, intestinal tissues were collected and stored at −80 °C.

Genomic DNA was extracted using the Marine Animal Tissue Genomic DNA Extraction Kit (Tiangen Biotech, Beijing, China). The integrity of the extracted DNA was assessed via 1% agarose gel electrophoresis, and the concentration and purity of the DNA were measured using a nucleic acid and protein analyzer (Biochrom Ltd, Cambridge, UK). All DNA samples were stored at −80 °C for subsequent analysis.

### 2.3. SSR-Seq Typing Process

Fifteen loci were retrieved from the existing literature for SSR typing [25], and primer sequences for these fifteen loci are summarized (Table 2). The standard genome was utilized as a template for amplification to fine-tune a multiplex touchdown PCR panel system. The PCR reaction mixture, with a total volume of 10 µL, consisted of 1 µL of 10 × buffer (Takara, Dalian, China), 0.07 µL of 5 U/µL HotStart Taq (Takara, Dalian, China), 1.2 µL of 2.5 mM dNTP, 0.6 µL of 25 mM MgCl_2_, 1 µL of template, 0.5 µL each of the forward and reverse primers, and 5.13 µL of ddH_2_O. The PCR amplification protocol comprised the following steps: initial pre-denaturation at 95 °C for 2 min, followed by 11 cycles of denaturation at 95 °C for 20 s, an initial annealing temperature of 63 °C for 40 s, and extension at 72 °C for 1 min. During the touchdown cycles, the annealing temperature was decreased by 0.5 °C per cycle. Subsequently, an additional 24 cycles were carried out at an annealing temperature of 65 °C for 30 s, resulting in a total of 35 cycles. Finally, a final extension was performed at 72 °C for 2 min, and the samples were stored at 4 °C. This method was employed to amplify the target fragments of the 23 parents and 150 offspring involved in the experiment. To ensure an equivalent amount of primers amplified at each site, the products of the same individual were combined. Subsequently, a specific tag sequence compatible with the Illumina platform was incorporated at the end of the library through PCR amplification using primers containing the index sequence (index primer). The PCR reaction mixture, with a volume of 10 µL, was composed of 1 µL of 10× buffer (Takara, Shanghai, China), 0.04 µL of 5 U/µL HotStart Taq (Takara, Shanghai, China), 0.8 µL of 2.5 mM dNTP, 0.2 µL of 25 mM MgCl_2_, 1 µL of template, 0.5 µL each of the forward and reverse index primers, and 5.96 µL of ddH_2_O. The PCR amplification steps were as follows: pre-denaturation at 95 °C for 2 min, 12 cycles of denaturation at 95 °C for 20 s, annealing at 60 °C for 40 s, and extension at 72 °C for 1 min. This was followed by a final extension at 72 °C for 2 min and storage at 4 °C. The index PCR amplification products of all samples were mixed in equal amounts, and the final FastTargetTM sequencing library was obtained through purification and recycling. The fragment length distribution of the library was verified using an Agilent 2100 bioanalyzer (Agilent Technologies, Santa Clara, CA, USA). After precisely quantifying the molar concentration of the library, high-throughput sequencing was carried out on the Illumina HiSeq platform in the 2 × 150/2 × 250 double-end sequencing mode.

### 2.4. Data Analysis

Genetic Diversity: Genetic diversity parameters, including the mean number of alleles (Na), effective number of alleles (Ne), observed heterozygosity (Ho), expected heterozygosity (He), polymorphic information content (PIC), and gene flow (Nm), were calculated using Popgene 3.4. The Hardy–Weinberg equilibrium (HWE) of SSR loci was tested with the R package pegas (V1.3).

Genetic Differentiation and Genetic Structure: Genetic differentiation and population structure were analyzed using FSTAT (V2.9.3.2, http://en.bio-soft.net/tree/FSTAT.html, accessed on 23 June 2022) and Arlequin (V3.5.2.2, https://cmpg.unibe.ch/software/arlequin35, accessed on 23 June 2022). F-statistics, including the inbreeding coefficient within populations (Fis), total inbreeding coefficient (Fit), and genetic differentiation coefficient among populations (Fst), were calculated for each locus, along with the Fis value for each population. Pairwise genetic distances between samples (Bruvo’s distance) were computed using the inbreedR polysat (V1.7–7) [26]. PCA was performed based on Nei’s genetic distances between subpopulations and Bruvo’s distances between individual samples. Phylogenetic trees for subpopulations and individual samples were constructed using the Neighbor-Joining (NJ) method. Outlier values were calculated with the R package ape (V5.7–1–1), and phylogenetic trees were visualized using ggtree. Molecular variance analysis (AMOVA) was conducted with the R package poppr (V2.9.4).

## 3. Results

### 3.1. Sequencing Results of the Offspring Population and SSR-Seq Genotyping Results

The sequencing data were aligned to the reference genome using Blast+ (V2.15.0, https://blast.ncbi.nlm.nih.gov/Blast.cgi, accessed on 25 June 2022), and the enrichment efficiency of the target fragments was evaluated. The enrichment efficiency for the offspring samples is summarized (Table 3).

**Figure 2:**
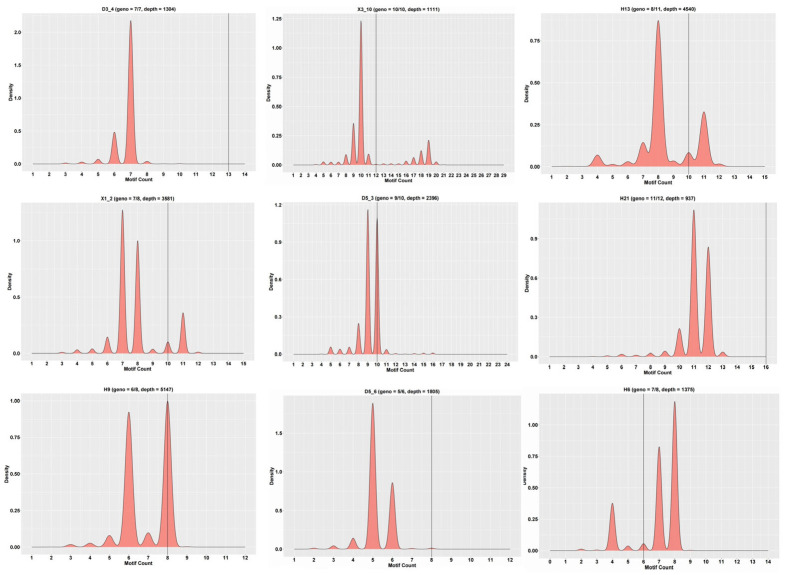
Examples of partial SSR loci typing in some offspring. Note: the horizontal coordinates represent the number of motifs, the vertical coordinates represent density, and the black vertical line in the middle represents the number of motifs in the reference sequence.

Using the sequencing data, the typing of 23 parents and 15 loci per parent was completed. Representative examples of SSR locus typing patterns in offspring cohorts are illustrated (Figure 2).

### 3.2. Genetic Diversity of Loci in the Offspring Population

Polymorphism statistics of the 15 SSR loci in the offspring population are summarized (Table 4). A total of 76 alleles were detected across the loci, with the number of alleles per locus ranging from 3 to 10. The average number of alleles (Na) was 5.067, while the average effective number of alleles (Ne) was 2.669. Among the loci, SSR3, SSR16, SSR18, SSR19, and SSRA22 exhibited the lowest Na, with only 3 alleles, whereas SSR1 had the highest Na, with 10 alleles. The observed heterozygosity (Ho) ranged from 0.240 to 0.820, with a mean value of 0.467, while the expected heterozygosity (He) ranged from 0.315 to 0.847, with an average of 0.570. The polymorphic information content (PIC) ranged from 0.285 to 0.829, with an average of 0.524. Seven loci (SSR2, SSR16, SSR17, SSR18, SSR19, SSR20, and SSRA9) were classified as moderately polymorphic loci with PIC values between 0.25 and 0.50. The remaining eight loci had PIC values greater than 0.50, indicating high polymorphism. Among the loci, SSR19 exhibited the lowest genetic diversity, with the smallest Ne (1.461), lowest He (0.315), and lowest PIC (0.285). Conversely, SSR1 demonstrated the highest genetic diversity, with the largest Ne (6.553), highest He (0.847), and highest PIC (0.829).

### 3.3. Genetic Diversity of the Three Offspring Populations

The genetic diversity parameters of the three offspring family populations across 15 SSR loci are summarized (Table 5). Notably, locus SSR19 exhibited no polymorphism in the C group. The observed number of alleles (Na) in the D and M groups was 4.200 and 4.733, respectively, which were both lower than the parental FP group (5.077) but higher than the C group (3.571). The effective number of alleles (Ne) in the D and M groups was 2.782 and 2.728, respectively, slightly lower than the parental FP group (2.816) but higher than the C group (2.211). The observed heterozygosity (Ho) in the D and M groups was 0.496 and 0.488, respectively, both lower than the parental FP group (0.522) but higher than the C group (0.447). The expected heterozygosity (He) for the D and M groups was 0.586 and 0.579, respectively, which were marginally lower than the parental FP group (0.595). Similarly, the polymorphic information content (PIC) values of the D and M groups were 0.530 and 0.531, respectively, indicating high levels of polymorphism. These values were lower than the parental FP group (0.546) but higher than the C group (0.438).

Among the 44 Hardy–Weinberg Equilibrium (HWE) tests conducted (14 loci for the C group and 15 loci each for the D and M groups), 12 loci significantly deviated from HWE, accounting for 31.11% of the total. Specifically, five loci in the D group, three loci in the C group, and four loci in the M group exhibited significant deviations from equilibrium. Notably, locus SSR2 showed significant deviations from HWE in all three groups, while loci SSR7, SSR16, and SSR18 deviated in two groups.

### 3.4. Analysis of Genetic Differentiation and Population Structure

The F-statistics for the 15 loci across the three offspring populations are summarized (Table 6). For Fis, five loci (SSR7, SSR16, SSR17, SSR19, and SSR20) exhibited negative values, with locus SSRA6 showing the highest positive value (0.574). Similarly, for Fit, four loci (SSR7, SSR16, SSR17, and SSR19) displayed negative values, while locus SSRA6 recorded the highest value (0.626). The Fst values ranged from 0.007 to 0.191, with an average of 0.062. Notably, 14 loci had Fst values below 0.15, except for locus SSR20. The Nm values varied between 1.056 and 37.201, with an average of 9.936.

The molecular variance analysis (AMOVA) revealed that 7.984% of the genetic variation occurred between populations, 10.922% between individuals within populations, and 81.093% within individuals (Table 7). The partitioning of variance components indicated that the majority of genetic variation was attributable to differences within individuals, highlighting that individual-level genetic variation was significantly greater than that between populations.

**Figure 3:**
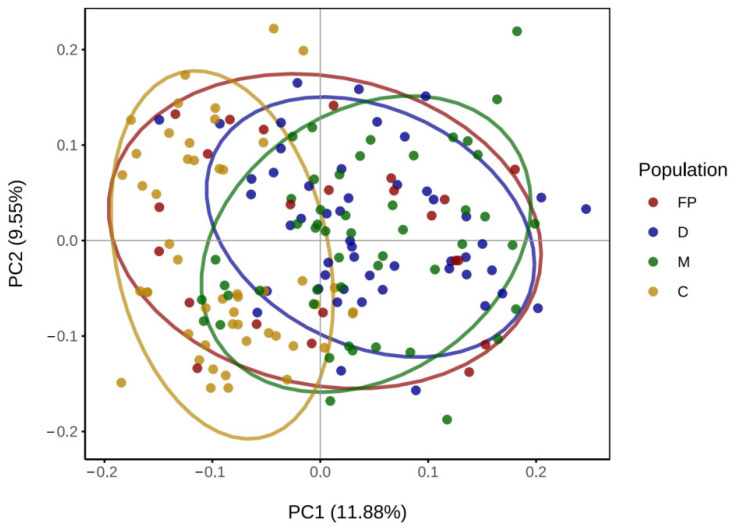
PCA results of all individuals in the parents and three offspring populations.

**Figure 4:**
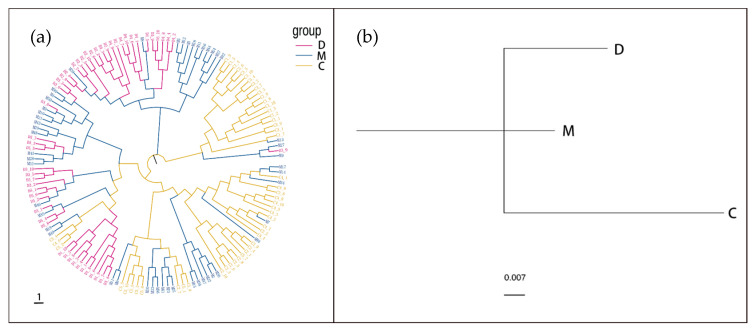
(**a**) Phylogenetic tree of all individuals in the three offspring populations. (**b**) An UPGMA clustering evolutionary tree of three offspring populations.

The PCA results are shown (Figure 3). Significant distributional overlap was observed among the parental FP, D, and M populations, while most individuals from the C population formed a separate cluster. However, when confidence ellipses were applied, the three offspring populations (D, M, and C) were found to cluster closely, indicating they belong to the same subgroup. The individual phylogenetic tree (Figure 4a) showed that individuals from the D and M populations were interspersed, while most individuals from the C population clustered together on the same branch. Nevertheless, no distinct geographic population branches were observed, which is consistent with the PCA results. The clustering tree of the three populations (Figure 4b) demonstrated that all three populations converged at the same node, indicating that they coexist as a single subgroup.

## 4. Discussion

### 4.1. Genetic Diversity Analysis of 15 Loci in Offspring Populations

Genetic diversity is a widely used metric for assessing the germplasm resources of a population and evaluating the degree of inbreeding. Key indicators for measuring genetic diversity include the number of alleles (Na/Ne), heterozygosity (Ho/He), and polymorphic information content (PIC). Higher levels of polymorphism reflect greater genetic diversity, which enhances the success and accuracy of parentage identification in family-based populations [27,28]. Specifically, loci are classified as highly polymorphic when PIC ≥ 0.5, moderately polymorphic when 0.25 ≤ PIC ≤ 0.5, and slightly polymorphic when PIC ≤ 0.25 [29]. In microsatellite sequence analysis, expected heterozygosity (He) and polymorphism information content (PIC) are important parameters for evaluating genetic variation and diversity within a population, with higher values indicating greater genetic diversity and enhanced adaptability to the environment [30,31].

In this study, the genetic diversity of the offspring populations derived from the FP showed a slight reduction compared to the parental FP. Across 15 loci, the average Na (5.067) and Ne (2.669) in the offspring were slightly lower than those of the parental FP (Na = 5.077, Ne = 2.816) [25]. Similarly, the average Ho (0.467) and He (0.570) in the offspring were lower than those of the parental FP (Ho = 0.522, He = 0.595) [25]. According to the criteria of Qin et al. [32], a population has high genetic diversity if its expected heterozygosity falls within the range of 0.5 to 0.8. All microsatellite loci in this study had PIC > 0.5, indicating that microsatellite loci of the *S. intermedius* demonstrated high genetic diversity. Although the average PIC value in the offspring populations remained within the highly polymorphic range, seven loci exhibited moderate polymorphism, indicating a decrease in heterozygosity at these loci. These results suggest that the genetic diversity of the offspring populations declined compared to the parental population, although the reduction was relatively minor. This reduction is likely due to the inclusion of some half-sib families within the offspring populations, which may have led to closer genetic relationships and lower levels of heterozygosity. Some loci showed deviations from the Hardy–Weinberg equilibrium, potentially due to the small population size and the multi-generational selection process employed in this study. Interestingly, the N group, which had the smallest parental genetic distance and the highest degree of inbreeding, exhibited the fewest deviations from Hardy–Weinberg equilibrium. This finding indicates that inbreeding was not the primary factor contributing to the observed deviations in the offspring populations.

### 4.2. Genetic Diversity Analysis of the Three Offspring Populations

In this study, the genetic diversity of offspring populations constructed based on parental genetic distances (D and C groups) and the mixed hybrid population (M group) was analyzed. Among these groups, the M group had the highest average number of alleles (Na), at 4.733, which was slightly lower than that of the parental FP group (5.000) [25]. The D and C groups exhibited lower Na values than the FP group, with the C group having the lowest, at 3.571. However, the D group showed the highest effective number of alleles (Ne), at 2.782, while the C group had the lowest, at 2.212. A reduction in the number of alleles at specific loci due to inbreeding is a common phenomenon in aquatic species. For example, Xing et al. [33] reported a significant reduction in the average number of alleles in three consecutive generations of artificially selected Crassostrea gigas populations, using 10 microsatellite markers in four multiplex PCR combinations. A similar trend was observed in Asian seabass after four generations of family-based selection [34].

The reduction in genetic diversity caused by inbreeding is not limited to the number of alleles but also affects observed heterozygosity (Ho) and expected heterozygosity (He). In this study, the average Ho for the D, C, and M groups was 0.496, 0.447, and 0.488, respectively, while the average He was 0.586, 0.487, and 0.579, respectively. Although these values were lower than those of the parental FP group (Ho = 0.522, He = 0.595) [25], the reductions in heterozygosity for the D and M groups were relatively minor compared to the N group, which showed a more pronounced decrease. These findings suggest that increasing the genetic distance between parents can effectively maintain the number of heterozygotes within offspring populations. The polymorphic information content (PIC) values for the D and M groups indicated high polymorphism, whereas the C group exhibited moderate polymorphism. Although the genetic diversity of all three offspring populations was reduced compared to the parental FP group, the decreases in the D and M groups were relatively small, while the N group showed a more significant reduction. In the D group, families were constructed using parental combinations with the highest genetic distances, maximizing the genetic diversity within the resulting single-family offspring population. The genetic diversity of the D group was comparable to that of the mixed hybrid M group, suggesting that the M group successfully preserved its genetic diversity. The higher PIC value in the M group compared to the family-based offspring populations may be attributed to the small-scale random mating and increased diversity of family lines within the M group. It is noteworthy that the offspring of group M also exhibited a relatively high level of genetic diversity, particularly in terms of expected heterozygosity (He) and polymorphic information content (PIC), which were similar to those of group D, which had a greater genetic distance. This may be attributed to the introduction of genetic material from multiple parents into group M, thereby enhancing gene flow between populations. Previous studies have demonstrated that gene flow plays a significant role in increasing genetic diversity [35,36], as it increases the opportunities for random recombination and broadens the genetic foundation of the population.

In conclusions, this study demonstrates that constructing families using parents with low genetic distances reduces genetic diversity in offspring populations. In contrast, using parents with greater genetic distances or employing mixed fertilization strategies effectively preserves the genetic diversity of offspring populations.

### 4.3. Genetic Differentiation and Genetic Structure of the Three Offspring Populations

The F-statistic parameter Fst is a key indicator for evaluating genetic differentiation among populations [37]. In this study, the Fst values among the three offspring populations indicated a low level of genetic differentiation. The Nm values, which measure genetic similarity between populations, were all greater than 1 and relatively high across the three offspring populations. This suggests substantial gene flow among the populations, which likely inhibited population differentiation and resulted in a high degree of genetic similarity among the populations. These findings are consistent with the fact that the offspring populations were constructed using parents derived from the same parental group. The average Fis value (0.120) and the average Fit value (0.171) further suggest limited genetic structure variation within the populations. Moreover, the AMOVA results confirmed that most of the genetic variation originated from differences among individuals, with relatively small differences observed between populations.

PCA (Figure 2) revealed that the D and M populations were closely related, exhibiting minimal genetic differentiation, while the C population showed noticeable genetic differentiation from the D and M populations. The individual phylogenetic tree (Figure 4a) provided further evidence of close genetic relationships among individuals and highlighted the degree of differentiation in the C population. These results suggest that breeding with parents of close genetic distance can effectively promote genetic differentiation. Genetic diversity in the M group remained lower than in the parental FP, despite having a population size comparable to natural populations [38]. This reduction likely originated from the FP group’s status as a selectively bred family line, leading to genetic purifying and inbreeding effects. In contrast, natural populations typically exhibit lower inbreeding levels due to stochastic gamete fusion during reproduction. In practical large-scale production settings, however, selecting larger numbers of parental individuals can achieve comparable genetic diversity outcomes. Both the individual phylogenetic tree and the population phylogenetic tree (Figure 4a) demonstrated that the three populations coexisted as parallel groups, indicating that the genetic differentiation of the C population was still in its early stages. The C population had not yet formed an independent population and remained part of the same subgroup as the D and M populations.

These population genetic structures can be effectively utilized for breeding programs. Genetic distance-assisted breeding is now widely applied in aquaculture. Wang et al. demonstrated that leveraging such information enables more effective prediction of hybrid advantage in fish [39]. Genomic selection models utilizing genetic markers (including genetic distance) significantly outperform traditional purebred-based models in accurately estimating genomic breeding values [40]. This approach provides a more effective strategy for disease-resistant breeding in rainbow trout, directly enhancing breeding efficiency.

## 5. Conclusions

In this study, we performed a comparative analysis of the genetic diversity of offspring populations constructed using three different parental genetic distances. The results demonstrated that genetic diversity decreased in the offspring populations, regardless of the genetic distance between parents. However, the reduction in genetic diversity was more pronounced in offspring produced by parents with closer genetic distances. Therefore, to preserve genetic diversity within the population and maintain appropriate genetic distances, the number of parental individuals can be increased for breeding purposes, large-scale gamete mixing can be implemented, and molecular biology techniques should be used to detect genetic distances between parents. This prevents excessively close genetic relationships and avoids inbreeding. This study provides a theoretical foundation for the selective breeding of *S. intermedius* and other aquatic species.

## Figures and Tables

**Table 1:** Parent composition and genetic distances of the three experimental populations.

Offspring Population ID	Female Parent ID	Male Parent ID	Genetic Distance Between Parents
D1	7	17	0.34297
D2	8	12	0.35118
D3	32	31	0.34743
D4	24	12	0.31827
D5	25	17	0.32214
Mean	—	—	0.33640
C1	23	18	0.12109
C2	13	20	0.12401
C3	21	18	0.12875
C4	6	20	0.13200
C5	27	28	0.14672
Mean	—	—	0.13051
M	2	3	0.11891~0.32214
	6	5	
	25	11	
	27	12	
	—	17	
	—	20	
	—	22	
	—	26	
	—	30	
	—	31	
Mean	—	—	0.29916

**Table 2:** Fifteen pairs of SSR-seq primer information.

No.	Locus	Repeat Motif	Number of Alleles	Primer Sequence (5′–3′)
1	SSR1	(CT)_12_	196~226	F: TCGTCATGAGATGGTCGCT R: CATTTTACCGTGGTGGGGTC
2	SSR2	(AG)_13_	179~187	F: CGCAGGATGCAGTGATACC R: ATTCCACCAGTATCCCAGCT
3	SSR3	(CT_)18_	136~180	F: GCGCTTAATCTTTGGATAATTG R: CTGTAGTCGCTCCGCATGT
4	SSR4	(AG)_12_	181~219	F: GGGAAGTTTTCCCCACTGAC R: TGTCCATAACGCCACATTCG
5	SSR7	(AC)_10_	199~213	F: TCCCATATGATTGCTCGTGC R: AGCATTCACCGCGAAACTG
6	SSR14	(AG)_10_	165~179	F: ATCCCAAACTACGTTCAACC R: GGCTGCCTAGTTGCATAAAT
7	SSR16	(CT)_16_	146~246	F: CCTTGGAATGAGAACTTGT R: ACCGATTTTACTTGACCTG
8	SSR17	(AT)_6_	231~237	F: CTGTTTGGATGAGTGGAAT R: TTTGAACGAGCTTGCCTT
9	SSR18	(TTGACT)_4_	112~130	F: GTCAGGTAGCTATATGTTC R: TGGTGATAAACATGTCAGAA
10	SSR19	(GCA)_8_	93~102	F: AGCTCCTAGGGTTCTTACC R: ACATGGGTGGAGAGGTG
11	SSR20	(GAA)_5_	147~156	F: CTAATAGCCCTATGCCGCGT R: ATACACCACACGATTCGCAC
12	SSRA6	(TGA)_6_	179~194	F: AAGCAGCCATTAAGGAAATG R: CAAGCAGGTTATCCGTTTCA
13	SSRA9	(TC)_9_	185~211	F: AAGCGAGCTTATGTCTAGTA R: CTAGAACCTTCATCAACTCT
14	SSRA10	(AC)_6_	148~168	F: CACGTATTTCGGATGGTGAC R: CTTATTATTAGCGCACGTCAT
15	SSRA22	(TCTG)_6_	186~202	F: GAAGAACCATGGACTTACTACA R: TGTTGTGAGAAAGGTAGCG

**Table 3:** Statistics of enrichment efficiency of progeny samples.

	Clean Reads	Raw Reads	Clean Reads Ratio	Q20	Q30
Average of all sampled offspring	45,295	53,518	0.85	100.00%	99.98%

**Table 4:** Genetic diversity indices of the 15 microsatellite loci.

Locus	*N*_a_	*N*_e_	*H*_o_	*H*_e_	*F*	*PIC*
SSR1	10	6.553	0.820	0.847	0.032	0.829
SSR2	4	1.576	0.298	0.365	0.185	0.337
SSR3	3	2.693	0.567	0.629	0.099	0.579
SSR4	9	3.280	0.380	0.695	0.453	0.664
SSR7	8	3.540	0.740	0.718	−0.031	0.673
SSR14	6	3.023	0.613	0.669	0.083	0.623
SSR16	3	1.743	0.473	0.426	−0.111	0.387
SSR17	4	1.975	0.567	0.494	−0.148	0.444
SSR18	3	2.144	0.252	0.534	0.528	0.445
SSR19	3	1.461	0.347	0.315	−0.099	0.285
SSR20	4	2.206	0.487	0.547	0.11	0.482
SSRA6	6	2.797	0.240	0.642	0.626	0.604
SSRA9	5	1.757	0.420	0.431	0.025	0.397
SSRA10	4	2.663	0.520	0.625	0.167	0.566
SSRA22	3	2.631	0.281	0.620	0.546	0.550
Mean	5.067	2.669	0.467	0.570	0.164	0.524

**Table 5:** Genetic diversity of the parental generation and three offspring populations at 15 microsatellite loci. Notes: ns. in accordance with Hardy–Weinberg Equilibrium; *. significant deviation from Hardy–Weinberg Equilibrium (*p* < 0.05); **. significant deviation from Hardy–Weinberg Equilibrium (*p* < 0.01).

Population	Locus	*N*_a_	*N*_e_	*H*_o_	*H*_e_	*PIC*	HWE
D	SSR1	9	6.624	0.842	0.849	0.831	ns
	SSR2	3	2.486	0.333	0.598	0.526	*
	SSR3	4	2.473	0.520	0.596	0.518	ns
	SSR4	7	3.748	0.480	0.733	0.704	ns
	SSR7	7	3.846	0.620	0.740	0.705	**
	SSR14	5	3.408	0.780	0.707	0.655	ns
	SSR16	3	1.644	0.420	0.392	0.356	ns
	SSR17	3	1.738	0.540	0.425	0.379	ns
	SSR18	3	1.971	0.180	0.493	0.405	ns
	SSR19	3	1.678	0.500	0.404	0.337	*
	SSR20	3	2.684	0.720	0.627	0.556	**
	SSRA6	4	2.641	0.340	0.621	0.572	ns
	SSRA9	3	1.691	0.400	0.409	0.361	ns
	SSRA10	3	2.145	0.420	0.534	0.462	**
	SSRA22	3	2.955	0.340	0.662	0.587	ns
	Mean	4.200	2.782	0.496	0.586	0.530	—
C	SSR1	6	4.909	0.778	0.796	0.766	ns
	SSR2	4	1.491	0.317	0.329	0.300	*
	SSR3	4	2.643	0.640	0.622	0.554	ns
	SSR4	4	2.859	0.340	0.650	0.583	ns
	SSR7	4	2.767	0.860	0.639	0.568	ns
	SSR14	3	2.256	0.440	0.557	0.494	**
	SSR16	3	1.744	0.500	0.427	0.383	ns
	SSR17	4	1.986	0.540	0.497	0.437	ns
	SSR18	2	1.777	0.229	0.437	0.342	**
	SSR20	2	1.173	0.160	0.147	0.136	ns
	SSRA6	3	1.545	0.060	0.353	0.323	ns
	SSRA9	4	1.660	0.420	0.398	0.369	ns
	SSRA10	4	2.632	0.680	0.620	0.576	ns
	SSRA22	3	1.525	0.300	0.344	0.300	ns
	Mean	3.571	2.212	0.447	0.487	0.438	—
M	SSR1	9	6.722	0.818	0.851	0.833	**
	SSR2	3	1.348	0.263	0.258	0.242	ns
	SSR3	4	2.234	0.540	0.552	0.494	ns
	SSR4	9	2.194	0.320	0.544	0.526	ns
	SSR7	7	3.802	0.740	0.737	0.695	**
	SSR14	5	2.921	0.620	0.658	0.608	ns
	SSR16	3	1.823	0.500	0.451	0.406	ns
	SSR17	3	2.121	0.620	0.529	0.468	*
	SSR18	3	2.679	0.366	0.627	0.548	**
	SSR19	3	1.818	0.540	0.450	0.403	ns
	SSR20	4	2.229	0.580	0.551	0.493	ns
	SSRA6	6	3.544	0.320	0.718	0.674	ns
	SSRA9	5	1.905	0.440	0.475	0.440	ns
	SSRA10	4	2.677	0.460	0.626	0.550	ns
	SSRA22	3	2.916	0.195	0.657	0.583	ns
	Mean	4.733	2.729	0.488	0.579	0.531	—
FP	SSR1	7	4.966	0.917	0.799	0.770	*
	SSR2	4	2.426	0.545	0.588	0.517	ns
	SSR3	5	3.061	0.656	0.673	0.622	ns
	SSR4	8	4.223	0.344	0.763	0.726	ns
	SSR7	8	3.977	0.750	0.749	0.712	*
	SSR14	6	3.606	0.781	0.723	0.681	ns
	SSR16	4	1.750	0.469	0.429	0.394	ns
	SSR17	4	2.181	0.656	0.542	0.487	ns
	SSR18	3	2.217	0.258	0.549	0.463	ns
	SSR19	3	1.724	0.344	0.420	0.354	ns
	SSR20	4	2.325	0.594	0.570	0.496	**
	SSRA6	5	2.711	0.161	0.631	0.588	ns
	SSRA9	5	1.438	0.313	0.305	0.292	ns
	SSRA10	4	2.131	0.625	0.531	0.481	ns
	SSRA22	3	2.186	0.296	0.543	0.478	**
	mean	5.077	2.816	0.522	0.595	0.546	—

**Table 6:** F-statistics of 15 microsatellite loci in the three offspring populations. Notes: ***N*_m_**. gene flow, which is estimated from ***F*_st_** = 0.25(1 − ***F*_st_**)/***F*_st_**.

Locus	*F*_is_	*F*_it_	*F*_st_	*N*_m_
SSR1	0.023	0.034	0.011	23.532
SSR2	0.229	0.292	0.082	2.795
SSR3	0.039	0.099	0.062	3.796
SSR4	0.409	0.453	0.076	3.053
SSR7	−0.049	−0.031	0.017	14.320
SSR14	0.042	0.083	0.043	5.554
SSR16	−0.118	−0.111	0.007	37.201
SSR17	−0.173	−0.148	0.021	11.717
SSR18	0.502	0.522	0.041	5.911
SSR19	−0.218	−0.099	0.097	2.317
SSR20	−0.101	0.110	0.191	1.056
SSRA6	0.574	0.626	0.122	1.796
SSRA9	0.017	0.025	0.009	28.947
SSRA10	0.124	0.167	0.050	4.768
SSRA22	0.497	0.547	0.099	2.275
Mean	0.120	0.171	0.062	9.936

**Table 7:** Result of AMOVA based on 15 microsatellite loci.

Source of Variation	*df*	Variance Components	Percentage of Variation	*p*-Value
Between population	2	0.5537	7.984	<0.01
Between individuals within population	147	0.7574	10.922	<0.01
Within individuals	150	5.6233	81.093	<0.01
Total	299	6.9344	100	—

## Data Availability

The original contributions presented in this study are included in the article; further inquiries can be directed to the corresponding authors.

## References

[1] Andrew N.L., Agatsuma Y., Ballesteros E., Bazhin A.G., Creaser E.P., Barnes D.K.A., Botsford L.W., Bradbury A., Campbell A., Dixon J.D. (2002). Status and management of world sea urchin fisheries. Oceanogr. Mar. Biol..

[2] Lawrence J.M., Lawrence A.L., Watts S.A. (2020). Ingestion, digestion, and digestibility of regular sea urchins. Dev. Aquac. Fish. Sci..

[3] Wang Z., Chang Y. (1997). Studies on hatching of Japanese sea urchin *Strongylocentrotus intermedius*. J. Fish. Sci. China.

[4] Agatsuma Y. (2013). *Strongylocentrotus intermedius*. Dev. Aquac. Fish. Sci..

[5] Zhou W., Sun J., Wang J., Du J. (2008). Current Status and Challenges of Sea Urchin Culture in China. J. Fish. Sci..

[6] Yu Y., Hilsdorf A.W.S., Zhou L., Lin Q., Gao Z.X. (2022). Genetics and molecular breeding in aquaculture animals. Front. Genet..

[7] Gheyas A., Basavaraju Y., McAndrew B., Penman D.J. (2007). Monitoring genetic diversity in a mass selection programme of common carp (*Cyprinus carpio*) using microsatellite markers. Aquaculture.

[8] Sui J., Luan S., Yang G., Chen X., Luo K., Gao Q., Wang J., Hu H., Kong J. (2018). Genetic diversity and population structure of a giant freshwater prawn (*Macrobrachium rosenbergii*) breeding nucleus in China. Aquac. Res..

[9] Ye Y., Ren W., Zhang S., Zhao L., Tang J., Hu L., Chen X. (2022). Genetic diversity of fish in aquaculture and of common carp (*Cyprinus carpio*) in Traditional Rice–Fish Coculture. Agriculture.

[10] Robisalmi A., Gunadi B., Alipin K., Artati D. (2023). Growth response and estimating heritability of synthetic base population (F0) of red tilapia (*Oreochromis* spp.) through family selection. IOP Conf. Ser. Earth Environ. Sci..

[11] Liu J., Kong J., Dai P., Yu Y., Meng X., Luo K., Cao B., Chen B., Gao H., Luan S. (2021). Development of SNP markers and verification analysis of relationship on family in *Litopenaeus vannamei*. Prog. Fish. Sci..

[12] Yáñez J.M., Bassini L.N., Filp M., Lhorente J.P., Ponzoni R.W., Neira R. (2014). Inbreeding and effective population size in a coho salmon (*Oncorhynchus kisutch*) breeding nucleus in Chile. Aquaculture.

[13] Cheng W., Xia R., Wang Q., Zeng K., Song W., Wei Q., Deng G., Cheng Y. (2020). Genetic diversity of *Siniperca chuatsi* in wild and cultivated populations. Freshw. Fish..

[14] Fu X., Liu J., Liu J. (2016). AFLP Analysis of Genetic Diversity in Five Selected Lines of abalone *Haliotis diversicolor* supertexta. J. Trop. Biol..

[15] Willoughby J.R., Fernandez N.B., Lamb M.C., Ivy J.A., Lacy R.C., DeWoody J.A. (2015). The impacts of inbreeding, drift and selection on genetic diversity in captive breeding populations. Mol. Ecol..

[16] Howard J.T., Pryce J.E., Baes C., Maltecca C. (2017). Invited review: Inbreeding in the genomics era: Inbreeding, inbreeding depression, and management of genomic variability. J. Dairy Sci..

[17] D’ambrosio J., Phocas F., Haffray P., Bestin A., Brard-Fudulea S., Poncet C., Quillet E., Dechamp N., Fraslin C., Charles M. (2019). Genome-wide estimates of genetic diversity, inbreeding and effective size of experimental and commercial Rainbow trout lines undergoing selective breeding. Genet. Sel. Evol..

[18] Würschum T., Zhu X., Zhao Y., Jiang Y., Reif J.C., Maurer H.P. (2023). Maximization through optimization? On the relationship between hybrid performance and parental genetic distance. Theor. Appl. Genet..

[19] Han Z., Han Q., Xia Y., Geng X., Du K., Yang J., Kang X. (2020). Construction of a breeding parent population of *Populus tomentosa* based on SSR genetic distance analysis. Sci. Rep..

[20] Wei J., Zhao L., Wu W., Luo K., Ye W., Fu Y., Chen C. (2016). Genetic characterization of *Oreochromis niloticus* (♀) × *Sarotherodon melanotheron* (♂) hybrid F2 and F3 by microsatellite analysis. S. China Fish. Sci..

[21] Chang Y., Zhang W., Zhao C., Song J. (2012). Estimates of heritabilities and genetic correlations for growth and gonad traits in the sea urchin *Strongylocentrotus intermedius*. Aquac. Res..

[22] Chang Y.Q., Zhang W.J., Leng X.F., Song J. (2015). *Strongylocentrotus intermedius* “Dajin”. China Fish..

[23] Wang Z.Z., Lu B.Y., Liu L. (2023). Progress and prospects of scientific and technological innovation in China’s aquaculture seed industry during the 13th Five-Year Plan period. J. Dalian Ocean Univ..

[24] Jiang H., Zhang W., Chen L., Liu J., Lv X., Wang Z., Liu L., Chang Y. (2020). Screening of disease-resistant families and analysis of growth performance in intermediate sea urchins. J. Dalian Ocean Univ..

[25] Liu L., Zhang W., Liu Y., Leng X., Ou F., Zang X., Li X., Ding J., Chang Y. (2023). Evaluation of the genetic diversity and genetic structure of multiple generation selection populations and unselected common population of sea urchin (*Strongylocentrotus intermedius*) using SSR-seq. J. Fish. China.

[26] Stoffel M.A., Esser M., Kardos M., Humble E., Nichols H., David P., Hoffman J.I. (2016). inbreedR: An R package for the analysis of inbreeding based on genetic markers. Methods Ecol. Evol..

[27] Jerry D.R., Preston N.P., Crocos P.J., Keys S., Meadows J.R., Li Y. (2004). Parentage determination of Kuruma shrimp *Penaeus* (*Marsupenaeus*) *japonicus* using microsatellite markers (Bate). Aquaculture.

[28] Zhang J., Ma W., Wang W., Gui J.F., Mei J. (2016). Parentage determination of yellow catfish (*Pelteobagrus Fulvidraco*) based on microsatellite DNA markers. Aquac. Int..

[29] Simonsen K.L., Churchill G.A., Aquadro C.F. (1995). Properties of statistical tests of neutrality for DNA polymorphism data. Genetics.

[30] Masatoshi N., Fumio T., Yoshio T. (1983). Accuracy of estimated phylogenetic trees from molecular data. J. Mol. Evol..

[31] Ke X., Liu J., Gao F., Cao J., Liu Z., Lu M. (2022). Analysis of genetic diversity among six dojo loach (*Misgurnus anguillicaudatus*) populations in the Pearl River Basin based on microsatellite and mitochondrial DNA markers. Aquac. Rep..

[32] Qin Y., Shi G., Sun Y. (2013). Evaluation of genetic diversity in *Pampus argenteus* using ssr markers. Genet. Mol. Res..

[33] Xing D., Li Q., Zhang X. (2017). Analysis of genetic diversity in mass selection lines of white-shell Pacific oyster (*Crassostrea gigas*) using microsatellite fluorescent multiplex PCR technique. J. Fish. China.

[34] Wong J., Sun F., Wang L., Yang Z.T., Wen Y.F., Pang H.Y., Lee M., Yeo S.T., Liang B., Chen K. (2023). Changes in genetic diversity of A*sian seabass in a 20-year breeding program. Aquaculture.

[35] Hans E., Nicolas G. (2016). Determinants of genetic diversity. Nat. Rev. Genet..

[36] David J.C., Margaret B., Craig M. (2018). Genetic Diversity and Conservation Units: Dealing With the Species-Population Continuum in the Age of Genomics. Front. Ecol. Evol..

[37] Slatkin M., Barton N.H. (1989). A comparison of three indirect methods for estimating average levels of gene flow. Evolution.

[38] Zhadan P.M., Vaschenko M.A., Permyakov P.A. (2021). Quantitative study of the behavior of two broadcast spawners, the sea urchins *Strongylocentrotus intermedius* and *Mesocentrotus nudus*, during mass spawning events in situ. PeerJ.

[39] Wang J., Xia D. (2002). Studies on fish heterosis with DNA fingerprinting. Aquac. Res..

[40] Vallejo R.L., Leeds T.D., Gao G., Parsons J.E., Martin K.E., Evenhuis J.P., Fragomeni B.O., Wiens G.D., Palti Y. (2017). Genomic selection models double the accuracy of predicted breeding values for bacterial cold water disease resistance compared to a traditional pedigree-based model in rainbow trout aquaculture. Genet. Sel. Evol..

